# A case of squamous cell carcinoma arising from a suprapubic cystostomy tract in a patient with spinal bifida: Immunohistochemical analysis and literature review

**DOI:** 10.1002/iju5.12554

**Published:** 2022-11-08

**Authors:** Harutake Sawazaki, Yosuke Kitamura, Atsushi Asano, Yuji Ito, Hitoshi Tsuda

**Affiliations:** ^1^ Department of Urology Tama‐Hokubu Medical Center Higashimurayama Japan; ^2^ Department of Urology National Defense Medical College Tokorozawa Japan; ^3^ Department of Pathology Tama‐Hokubu Medical Center Higashimurayama Japan; ^4^ Department of Basic Pathology National Defense Medical College Tokorozawa Japan

**Keywords:** biomarker, immunohistochemical analysis, spina bifida, squamous cell carcinoma, suprapubic cystostomy tract

## Abstract

**Introduction:**

Squamous cell carcinoma arising from a suprapubic cystostomy tract is a rare complication of an indwelling catheter and is caused by long‐term inflammation and mechanical irritation. Prognosis is relatively poor. Biomarkers in the cancer pathway have not been investigated.

**Case presentation:**

A 61‐year‐old woman with a 34‐year history of suprapubic catheter placement presented with a rapidly growing elevated lesion around the cystostomy site. Tumor biopsy confirmed squamous cell carcinoma. Local excision with partial cystectomy was performed. Multiple metastases were identified 5 months later. The patient died 14 months after the initial treatment. Immunohistochemical analysis of the resected specimen revealed alterations in vascular endothelial growth factor, epidermal growth factor receptor, cyclooxygenase‐2, and Ki‐67.

**Conclusion:**

We encountered a case of squamous cell carcinoma arising from a suprapubic cystostomy tract. Immunohistochemical analysis revealed activation of multiple carcinogenic pathways in cancer cells, including those for angiogenesis, signal transduction by epidermal growth factor receptor, inflammation, and cell proliferation.

**Abbreviations & Acronyms**
COX‐2: cyclooxygenase‐2
EGFR: epidermal growth factor receptor
IHC: immunohistochemical analysis
SCC: squamous cell carcinoma
SCT: suprapubic cystostomy tract
VEGF: vascular endothelial growth factor

**Keynote message**

We encountered a patient with squamous cell carcinoma arising from a suprapubic cystostomy tract who died 14 months after initial treatment. Immunohistochemical analysis revealed activation of multiple carcinogenic pathways in the cancer cells, including those for angiogenesis, signal transduction by EGFR, inflammation, and cell proliferation. Further research is needed to understand the biology of squamous cell carcinoma arising from a suprapubic cystostomy tract and to establish a treatment strategy.

## Introduction

SCC arising from a SCT is extremely rare and very few cases have been reported.^1^ Although bladder SCC can arise in the setting of long‐term bladder catheterization and chronic inflammation,^2^ SCC of the suprapubic tract arises from chronic irritation by the indwelling catheter and long‐term inflammation of the tract and surrounding skin^2^, ^3^ and the prognosis is relatively poor.^4^ Biomarkers of the cancer‐related pathways in this rare entity have not been investigated. In this report, we describe a case of SCC arising from an SCT. We performed IHC of various biomarkers involved in an angiogenesis marker VEGF; a signal transduction marker EGFR; an inflammatory marker COX‐2; a cell proliferation marker Ki‐67; cell cycle regulatory markers p53, p21, and cyclin E and an apoptotic marker Bcl‐2 in resected specimen.

## Case presentation

**Fig. 1:**
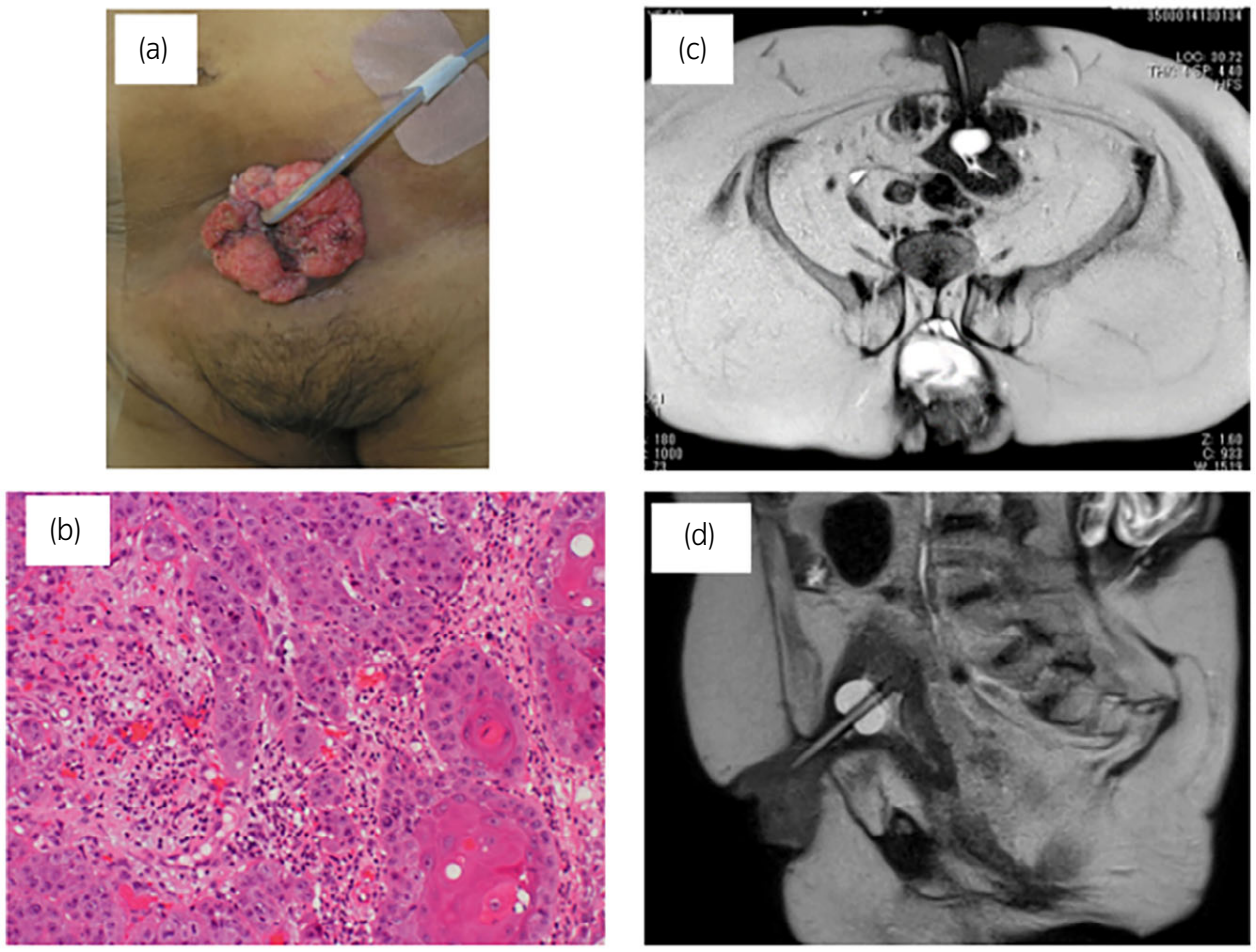
SCC arising from a SCT. (a) Clinical photograph showing an elevated lesion around the suprapubic cystostomy. (b) Tumor biopsy demonstrated a well‐differentiated SCC. (c, d). Magnetic resonance images showing that the tumor involved the bladder wall along the site of the catheter.

**Fig. 2:**
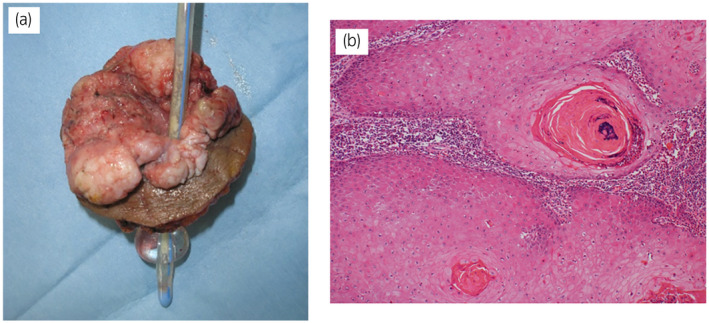
(a) Clinical photograph of the en bloc‐resected specimen, including the skin lesion, muscle, suprapubic catheter, and urinary bladder. (b) Micrograph demonstrating well‐differentiated SCC composed of well‐defined intercellular bridges with keratinization in individual cells and keratin pearls.

A 61‐year‐old Japanese woman with paraplegia secondary to spina bifida and no history of cigarette smoking was referred to our hospital for investigation of a rapidly growing elevated lesion around a cystostomy site (Fig. 1a). A suprapubic cystostomy and colostomy had been performed at the age of 27 years. Tumor biopsy confirmed well‐differentiated SCC (Fig. 1b). Magnetic resonance imaging revealed a tumor along the catheter involving the bladder wall (Fig. 1c,d). No lymph node or visceral metastasis was evident on computed tomography. The serum SCC level was elevated to 19.4 ng/mL. Local excision with partial cystectomy was performed (Fig. 2a). The abdominal wall defect was closed primarily and a new suprapubic catheter was inserted in the bladder. Pathological examination confirmed SCC arising from SCT (Fig. 2b). Five months after surgery, a right pelvic lymph node metastasis and subcutaneous metastasis in the right inguinal region were identified on computed tomography. The right inguinal metastasis strongly compressed the right femoral vein and the right lower extremity became edematous. The pelvic lymph nodes were dissected, the subcutaneous metastasis was resected, and the right lower limb was amputated. Adjuvant chemotherapy (cisplatin 75 mg/m^2^ on day 1 and doxorubicin 40 mg/m^2^ on day 2) was administered. After 2 courses of chemotherapy, new subcutaneous metastases were found in the pelvis and right abdominal wall, and lymph node metastasis was detected in the right axilla. Chemotherapy (irinotecan 100 mg/m^2^ on days 1, 8, 15, and 22) was administered. The metastases progressed after 2 courses of irinotecan. Multiple new subcutaneous metastases appeared in the right chest wall and right thigh. She died 14 months after initial treatment.

**Fig. 3:**
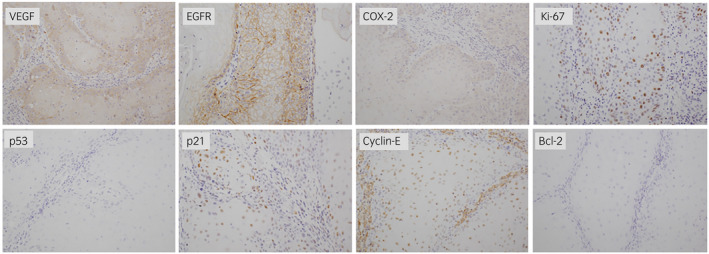
Results of IHC. The expression level of each biomarker was evaluated according to the staining intensity and proportion of positive cells in the total cell population. The antibodies used for the analyses were as follows: rabbit anti‐VEGF polyclonal antibody (dilution 1:50, Zymed, South San Francisco, CA, USA), mouse anti‐EGFR monoclonal antibody (ready to use, 2‐18C9; Dako Corp, Carpinteria, CA, USA), mouse anti‐COX‐2 monoclonal antibody (dilution 1:100, CX‐294, 1:100; Dako Corp), rabbit anti‐Ki‐67 monoclonal antibody (ready to use, SP6; Nichirei, Tokyo, Japan), mouse anti‐p53 monoclonal antibody (dilution 1:50, DO‐7; Dako Corp), mouse anti‐p21 monoclonal antibody (dilution 1:50, SX118; Dako Corp), mouse anti‐cyclin E monoclonal antibody (ready to use, ME12; Diagnostic BioSystems, Pleasanton, CA, USA), and mouse anti‐Bcl‐2 monoclonal antibody (dilution 1:100, clone 124; Dako Corp).

IHC was performed for VEGF, EGFR, COX‐2, Ki‐67, p53, p21, cyclin E, and Bcl‐2. The antibodies used for the analyses are described in Figure 3. The expression level of each biomarker was evaluated according to the staining intensity and proportion of positive cells in the total cell population. The immunoreactivity of each biomarker was classified as altered or not altered as reported previously.^5^, ^6^, ^7^, ^8^, ^9^ VEGF expression was scored by assigning an intensity score and a proportion score. The intensity score represented the average intensity of positive cells (0, none; 1, weak; 2, intermediate; 3, strong). The proportion score represented the estimated proportion of cells that stained positive (0, 0–24%; 1, 25–49%; 2, 50–74%, and 3, 75–100%). The intensity and proportion scores were then combined (0–6). VEGF expression was considered altered if the combined score was ≥3, EGFR expression as altered if >25% of tumor cells stained positive, COX‐2 expression as altered if >20% of tumor cells stained positive, Ki‐67 expression as altered if the Ki‐67 labeling index was >20%, p53 expression as altered if ≥10% cells stained positive, p21 expression as altered if <10% cells stained positive, cyclin E expression as altered if <30% cells stained positive, and Bcl‐2 expression as altered if >20% cells stained positive.

In our patient, the combined score for VEGF was 5. The percentage of EGFR‐positive cells was 100% and that of COX‐2‐positive cells was 30%. The Ki‐67 labeling index was 50%. The percentage of p53‐positive cells was 5%, that of p21‐positive cells was 40%, and that of cyclin E‐positive cells was 60%. There were no Bcl‐2‐positive cells (Fig. 3). Therefore, the immunoreactivity of VEGF, EGFR, COX‐2, and Ki‐67 was considered altered and that of p53, p21, cyclin E, and Bcl‐2 was considered not altered.

## Discussion

SCC arising from SCT is managed primarily by surgical intervention, but radiotherapy is used in patients who are not suitable for surgery and/or have metastatic disease. The prognosis is relatively poor because of recurrence or complications from treatment. Recurrence is usually within a year following treatment.^10^ There are only 12 cases reported in the literature,^1^, ^2^, ^3^, ^4^, ^10^, ^11^, ^12^, ^13^, ^14^, ^15^, ^16^ which are summarized in Table 1. The treatments used were local excision (*n* = 7), radiation (*n* = 4), and both local excision and radiation (*n* = 1). Five patients were still alive at a mean of 9.4 months after initial treatment and four had died (three of the disease) at a mean of 6 months after starting treatment and one of pneumonia after 5 months. The outcomes in the other three patients are unknown.

**Table 1:** Details of the 12 cases reported in the literature and our case

Author, published year	Age, year	Gender	Indication for cystostomy	Duration of cystostomy, year	Bladder involvement	Metastasis	Treatment modality	Chemotherapy	Prognosis
Stroumbakis N, 1993	80	Male	Urethral stricture	5	None	None	Preoperative radiation (2000 rad), mass excision, and partial cystectomy	None	Unknown
Strokes H, 1995	50	Male	Paraplegia	25	Yes	None	Mass excision and total cystectomy	None	Dead of disease (8 months)
Schaafsma RJH, 1999	63	Male	Paraplegia	37	None	None	Mass excision and partial cystectomy	None	Dead of pneumonia (5 months)
Gupta NP, 2000	40	Male	Urethral stricture	20	Yes	None	Mass excision and total cystectomy	None	Alive (3 months)
Ito H, 2011	58	Male	Paraplegia	35	Unknown	Lymph node metastases	Palliative radiation 56 Gy	None	Alive (6 months)
Chung JM, 2013	56	Male	Urethral stricture	9	Yes	None	Radiation (dose not described)	None	Dead of disease (6 months)
Massaro PA, 2014	55	Male	Paraplegia	38	Yes	None	Mass excision and partial cystectomy	None	Unknown
Massaro PA, 2014	85	Female	Idiopathic urinary retention	0.75	Yes	None	Mass excision and partial cystectomy	None	Unknown
Ranjan N, 2015	68	Male	Urethral stricture	20	Yes	None	Palliative radiation (dose not described)	None	Dead of disease (4 months)
Zhang X, 2015	61	Male	Paraplegia	28	Yes	None	Radiation 60 Gy	None	Alive (2 years)
Suramaniam S, 2017	88	Male	Urethral stricture	25	None	None	Mass excision and partial cystectomy	None	Alive (6 months)
Khadhouri S, 2018	53	Male	Paraplegia	20	Yes	None	Mass excision and partial cystectomy	None	Alive (8 months)
Our case	61	Female	Paraplegia	34	Yes	None	Mass excision and partial cystectomy	Yes	Dead of disease (14 months)

Chemotherapy is considered for patients with metastatic disease^15^ but was not administered in any of the previous cases. In our case, cisplatin and doxorubicin were administered as first‐line agents^17^ and irinotecan as second‐line therapy^18^ but neither was effective. Elevated EGFR expression has been identified in advanced cutaneous SCC, and an anti‐EGFR antibody, such as cetuximab, administered in combination with platinum‐based chemotherapy has been reported to be effective.^19^ In view of the immunoreactivity of EGFR identified in our case, addition of cetuximab to the chemotherapy regimen may have a beneficial effect in this type of cancer.

## Conclusion

We encountered a case of SCC arising from an SCT. IHC revealed activation of multiple carcinogenic pathways in the cancer cells, including those for angiogenesis, signal transduction by EGFR, inflammation, and cell proliferation.

## Author contributions

Harutake Sawazaki: Investigation, Methodology, Writing – original draft. Yosuke Kitamura: Supervision. Atsushi Asano: Supervision. Yuji Ito: Investigation; methodology. Hitoshi Tsuda: Investigation; methodology; supervision.

## Conflict of interest

The authors declare no conflict of interest.

## Approval of the research protocol by an Institutional Reviewer Board

The study was approved by the relevant ethics committee.

## Informed consent

Written informed consent was obtained from the patient's family.

## Registry and the Registration No. of the study/trial

Not applicable.
